# Radiotherapy for gastric lymphoma: a planning study of 3D conformal radiotherapy, the half-beam method, and intensity-modulated radiotherapy

**DOI:** 10.1093/jrr/rru052

**Published:** 2014-08-25

**Authors:** Koji Inaba, Hiroyuki Okamoto, Akihisa Wakita, Satoshi Nakamura, Kazuma Kobayashi, Ken Harada, Mayuka Kitaguchi, Shuhei Sekii, Kana Takahashi, Kotaro Yoshio, Naoya Murakami, Madoka Morota, Yoshinori Ito, Minako Sumi, Takashi Uno, Jun Itami

**Affiliations:** 1 Department of Radiation Oncology, National Cancer Center Hospital, 5-1-1 Tsukiji, Chuo-ku, Tokyo 104-0045, Japan; 2 Diagnostic Radiology and Radiation Oncology, Graduate School of Medicine, Chiba University, 1-8-1 Inohana, Chuo-ku, Chiba City, Chiba 260-8670, Japan

**Keywords:** primary gastric lymphoma, radiotherapy, IMRT, half-field method, planning study

## Abstract

During radiotherapy for gastric lymphoma, it is difficult to protect the liver and kidneys in cases where there is considerable overlap between these organs and the target volume. This study was conducted to compare the three radiotherapy planning techniques of four-fields 3D conformal radiotherapy (3DCRT), half-field radiotherapy (the half-beam method) and intensity-modulated radiotherapy (IMRT) used to treat primary gastric lymphoma in which the planning target volume (PTV) had a large overlap with the left kidney. A total of 17 patients with gastric diffuse large B-cell lymphoma (DLBCL) were included. In DLBCL, immunochemotherapy (Rituximab + CHOP) was followed by radiotherapy of 40 Gy to the whole stomach and peri-gastric lymph nodes. 3DCRT, the half-field method, and IMRT were compared with respect to the dose–volume histogram (DVH) parameters and generalized equivalent uniform dose (gEUD) to the kidneys, liver and PTV. The mean dose and gEUD for 3DCRT was higher than for IMRT and the half-beam method in the left kidney and both kidneys. The mean dose and gEUD of the left kidney was 2117 cGy and 2224 cGy for 3DCRT, 1520 cGy and 1637 cGy for IMRT, and 1100 cGy and 1357 cGy for the half-beam method, respectively. The mean dose and gEUD of both kidneys was 1335 cGy and 1559 cGy for 3DCRT, 1184 cGy and 1311 cGy for IMRT, and 700 cGy and 937 cGy for the half-beam method, respectively. Dose–volume histograms (DVHs) of the liver revealed a larger volume was irradiated in the dose range <25 Gy with 3DCRT, while the half-beam method irradiated a larger volume of liver with the higher dose range (>25 Gy). IMRT and the half-beam method had the advantages of dose reduction for the kidneys and liver.

## INTRODUCTION

Primary gastric lymphomas are mainly consists of mucosa-associated lymphoid tissue (MALT) lymphoma and diffuse large B-cell lymphoma (DLBCL). While standard treatment of gastric MALT lymphoma refractory to *Helicobacter pylori* (HP) sterilization is radiotherapy of ∼30 Gy with conventional fractionation [1, 2], localized gastric DLBCL is treated with immunochemotherapy and ensuing radiotherapy of ∼30–40 Gy with conventional fractionation [3, 4]. The clinical target volume (CTV) for gastric lymphoma is the whole stomach and neighboring peri-gastric lymph node stations. The stomach shows physiological motions due to respiration and peristalsis, which necessitates large margins being added to the CTV to set up the internal target volume (ITV). During radiotherapy of the stomach, the main organs at risk (OARs) are the kidneys and liver. Meticulous attention should be paid to kidney and liver tolerances because they are relatively sensitive to radiation. The tolerance dose is expressed as TD_5/5_ and TD_50/5_, indicating the doses at which late morbidity is seen in 5% and 50% respectively in 5 years. TD_5/5_ and TD_50/5_ for whole kidney irradiation are 23 Gy and 28 Gy, respectively. Also, TD_5/5_ and TD_50/5_ for whole liver irradiation have been reported to be 30 Gy and 40 Gy, respectively [5]. Radiation effects to the kidneys and liver were recently summarized in the QUANTEC study [6, 7, 8].

There are numerous reports about treatment planning for the abdominal region, e.g. gastric carcinoma [9] or pancreas carcinoma and/or bile duct malignancies [10, 11], and many reports indicate the advantages of intensity-modulated radiotherapy (IMRT). For gastric lymphoma, however, there are few reports about radiation treatment planning. In one such report, Della *et al.* classified gastric lymphoma into three types according to the amount of overlap between the kidney and the planning target volume (PTV). In cases with a large overlap between the kidney and the PTV, it is difficult to protect the kidney when using radiation of AP/PA opposing fields; in these cases, 3D conformal radiation therapy (3DCRT) is more advantageous. Use of IMRT might lead to further improvement for the left kidney and liver doses [12]. Additionally, Ringash *et al.* reported a five-fields technique for postoperative radiotherapy of gastric cancer. In this technique, the target volume is divided at the isocenter, which is typically placed at the level of the upper end of the left kidney. The volume superior to this isocenter is treated with AP/PA half-beam opposing fields, wheras the inferior volume is irradiated with an anterior and two half-beam wedged lateral fields. The resulting anterior fields irradiate the superior and inferior volumes with a single isocenter. A junctional move of 1 cm superiorly is planned after 10 fractions. This method was used to treat 20 patients, and acute toxicity was reduced to 25%, which is lower than the 41% observed in INT0116 [13]. INT0116 was a phase III study of chemoradiotherapy (fluorouracil plus leucovorin and 45 Gy/25 fractions to the tumor bed, regional nodes and 2 cm beyond the proximal and distal margins of resection) after surgery versus surgery alone for adenocarcinoma of the stomach or gastroesophageal junction [14]. We modified the method of Ringash *et al.* by deleting the lower half of the anterior field so as to reduce the dose to the left kidney. This half-beam method uses superior AP/PA half-beam fields and inferior LR/RL half-beam fields.

The current planning study was conducted on difficult cases with a large overlap between the kidney and the PTV, comparing the three planning techniques of 3DCRT, the half-beam method and IMRT.

## MATERIALS AND METHODS

The radiation oncology database was searched for gastric DLBCL treated with radiotherapy of ∼40 Gy/20 fractions from 1 January 2000 to 31 December 2012, whose 3D CT data was available for radiation planning. Eligible patients had an overlap of >50% between the left kidney and the PTV in an AP projection in digitally reconstructed radiography (DRR).

Patients were instructed to fast before treatment. In some patients, scopolamine butylbromide was used to suppress peristalsis. For X-ray simulation, a small amount of barium was swallowed to examine the degree of peristalsis. The CTV was defined as the whole stomach plus the neighboring peri-gastric lymph nodes. Planning CT was obtained in shallow exhale and inhale phases, and CTVs in both phases were fused and margins of peristalsis were added to obtain the ITV. The PTV was expanded 1 cm in all directions from the ITV to cover inter- and intra-fractional gastric motion and the set-up margin. On-board imaging (OBI) was used to confirm the fields covered the target sufficiently with a small amount of barium during treatment. During radiotherapy planning, the kidneys were contoured as the renal parenchyma excluding renal calyces. The three plans of 3DCRT, the half-beam method and IMRT were constructed using beam data generated by 15-MV X-rays from a linear accelerator (Clinac iX Linear accelerator, Varian, Palo Alto, CA). In 3DCRT, AP/PA or oblique fields and LR/RL fields or oblique fields were set up that covered the PTV adequately. In the half-beam method, the isocenter was set at the upper end of the left kidney, and the upper half of the PTV was treated using half-beam AP/PA or oblique fields with the reference point set to the central point of the field, while the lower half was irradiated with half-beam RL/LR or oblique lateral fields with the reference point set to the central point of the field. The junction was moved once a week. In the 3DCRT and half-beam methods, 40 Gy was applied to the isocenter. Nine fields at gantry angles of 0°, 35°, 60°, 110°, 165°, 210°, 290°, 315° and 340° were used in the IMRT treatment plan. Doses to the kidney and the liver were reduced to as low as possible, and the dose prescription in the IMRT treatment plan was defined by 95% of the PTV (D95) being irradiated to an equal dose to the 3DCRT planning.

Dose–volume histograms (DVHs) of the PTV, the liver, the right kidney, left kidney and both kidneys were analyzed. The mean dose and generalized equivalent uniform dose (gEUD) were compared between the three treatment plans. The gEUD is the uniform whole-organ dose that would cause equivalent biologic effect to the inhomogeneous dose distribution in the relevant organ. The gEUD can be obtained using the formula

$$\text{gEUD}={\left(\sum_{i}v_{i}D_{i}^{a}\right)}^{1/a}$$

[15], where *v_i_* is the fractional organ volume receiving a dose *D_i_* and *a* is a tissue-specific parameter that represents the volume effect.

In the statistical analysis, each parameter was analyzed by non-parametric comparison. A *P* value < 0.0167 was considered significant when two of the three plans were evaluated (multiple comparison, Bonferroni method). Our institutional review board (the National Cancer Center Institutional Review Board) approved this study, and treatment was carried out with written informed consent.

## RESULTS

**Fig. 1.**
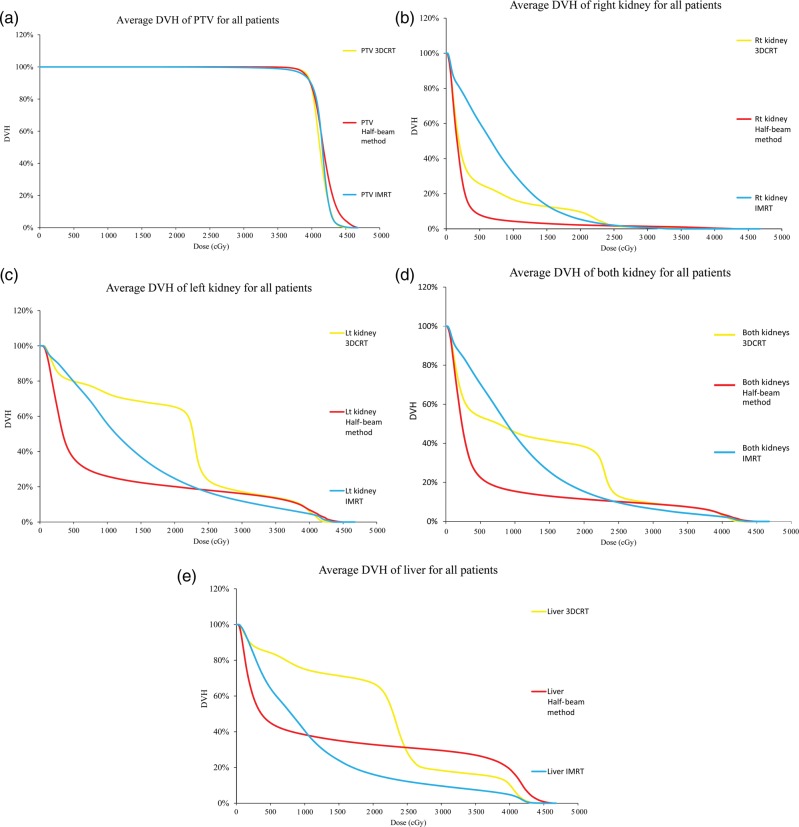
Average DVH comparing 3DCRT, the half-beam method and IMRT. (a) DVH of PTV, (b) DVH of the right kidney, (c) DVH of the left kidney, (d) DVH of both kidneys, and (e) DVH of the liver.

A total of 92 patients with gastric DLBCL underwent post-chemotherapy radiation therapy in the study period; 17 of the 92 patients (18.5%) were classified as ‘difficult’ cases with a large overlap of the left kidney and the PTV, and were thus included in this study. Average DVHs for the three plans are shown in Fig. 1. The mean dose and the gEUD of the three plans are summarized in Table 1.

**Table 1.** Mean doses and gEUDs of right, left and both kidneys and liver according to 3DCRT, the half-beam method and IMRT

	3DCRT (mean)	Half-beam method (mean)	IMRT (mean)	*P* value IMRT vs half-beam method	*P* value half-beam method vs 3DCRT	*P* value IMRT vs 3DCRT
Rt kidney						
mean dose (cGy)	519	283	835	<0.0001*	0.021	0.0012*
gEUD (cGy)	622	344	913	<0.0001*	0.0192	0.0043*
Lt kidney						
mean dose (cGy)	2117	1100	1520	0.0175	0.0003*	0.023
gEUD (cGy)	2224	1357	1637	0.2025	0.0017*	0.0192
Both kidneys						
mean dose (cGy)	1335	700	1184	0.0002*	<0.0001*	0.2025
gEUD (cGy)	1559	937	1311	0.0034*	0.0002*	0.0915
Liver						
mean dose (cGy)	2115	1520	1135	0.0007*	0.0003*	<0.0001*
gEUD (cGy)	2656	2746	1976	<0.0001*	0.2856	<0.0001*

*P < 0.0167 considered to have a significant difference.

Comparing the DVHs in the dose range <15 Gy, an increasing volume of the right kidney was irradiated by the half-beam method, 3DCRT and IMRT (in that order). In the dose range >25 Gy, the DVHs of the three plans converged with one another. The resulting mean dose and gEUD of the right kidney for IMRT was higher than for 3DCRT and the half-beam method with statistical significance. The mean dose for IMRT was 835 cGy, for 3DCRT was 519 cGy (*P* = 0.0012) and for the half-beam method was 283 cGy (*P* < 0.0001). The gEUD for IMRT was 913 cGy, for 3DCRT was 622 cGy (*P* = 0.0043) and for the half-beam method was 344 cGy (*P* < 0.0001).

Similarly, in the dose range <25 Gy, an increasing volume of the left kidney was irradiated using the half-beam method, IMRT, and 3DCRT (in that order). In the higher dose range (>25 Gy), the DVHs of the three plans showed no difference. The resulting mean dose and the gEUD for 3DCRT was higher than for IMRT and the half-beam method, with the difference between the half-beam method and 3DCRT reaching statistical significance. The mean dose for 3DCRT was 2117 cGy, for IMRT was 1520 cGy (*P* = 0.023) and for the half-beam method was 1100 cGy (*P* = 0.0003). The gEUD for 3DCRT was 2224 cGy, for IMRT was 1637 cGy (*P* = 0.0192) and for the half-beam method was 1357 cGy (*P* = 0.0017).

Regarding the DVHs for both kidneys, in the low dose range (<10 Gy) an increasing volume of both kidneys was irradiated using the half-beam method, 3DCRT and IMRT (in that order), while in the moderate dose range (between 10 Gy and 25 Gy) an increasing volume of both kidneys was irradiated using the half-beam method, IMRT and 3DCRT (in that order); for >25 Gy the DVHs of the three plans converged with one another. The resulting mean dose and the gEUD for both kidneys using 3DCRT and IMRT was higher than using the half-beam method with statistical significance. The mean dose for the half-beam method was 700 cGy, for 3DCRT was 1335 cGy (*P* < 0.0001) and for IMRT was 1184 cGy (*P* = 0.0002). The gEUD using the half-beam method was 937 cGy, using 3DCRT was 1559 cGy (*P* = 0.0002) and using IMRT was 1311 cGy (*P* = 0.0034).

In the DVHs of the liver, a larger volume was irradiated using 3DCRT in the dose range <25 Gy, while the half-beam method irradiated a larger volume of the liver in the higher dose range. Although the mean dose of the liver was largest using 3DCRT (the mean dose using 3DCRT was 2115 cGy, using the half-beam method was 1520 cGy (*P* = 0.0003) and using IMRT was 1135 cGy (*P* < 0.0001)), the gEUD was largest using the half-beam method because the half-beam method exposed a larger volume of the liver in the dose range >25 Gy (the gEUD using the half-beam method was 2746 cGy, using 3DCRT was 2656 cGy (*P* = 0.2856) and using IMRT was 1976 cGy (*P* < 0.0001)).

## DISCUSSION

In radiotherapy for gastric lymphoma, cases with a small overlap between the left kidney and the PTV are easy for radiotherapy planning, but for cases with a large overlap between them, OAR dose reduction is very difficult. We conducted this study to compare dose distributions for 3DCRT, the half-beam method and IMRT to establish the benefits of each radiation method in difficult cases where there is a large overlap between the left kidney and the PTV. The half-beam method and IMRT showed a great advantage over 3DCRT in reducing the doses to the left kidney and liver.

This planning study revealed that the half-beam method and IMRT were appropriate treatment plans for cases with a large overlap between the left kidney and the PTV in terms of reducing the doses to the left kidney and liver. The half-beam method irradiated the bilateral kidneys with the lowest dose, which may be quite important for the possible future administration of chemotherapy. However, the half-beam method had a disadvantage in that the high-dose region of the liver was larger. Meanwhile, IMRT had a tendency for the mean dose to the kidney being larger than for the half-beam method. The tolerance dose of the kidneys is lower than that of the liver; therefore, the half-beam method seems to be preferable to IMRT. However, the dose distribution for IMRT can be altered by changing the setting of the priority parameters of the OARs during planning; thus, it was difficult to decide whether IMRT or the half-beam method was more suitable. It seems advisable to make plans both using the half-beam method and IMRT and then to compare them with respect to the DVHs to decide which plan will be more suitable. Lately, localized DLBCL has sometimes been treated by R-CHOP alone; therefore, these difficult cases could be candidates for immune-chemotherapy alone. In the QUANTEC, the tolerance dose for both kidneys is reported to be ∼15–18 Gy in mean dose, and the tolerance dose for the normal liver is reported to be ∼30–32 Gy in mean dose [8]. However, there have been some reports showing that late renal toxicity occurs even under 15–18 Gy in long-term follow-up study of patients undergoing total-body irradiation (TBI) [16, 17, 18]. Cheng *et al*. reported that the dose associated with a 5% risk of kidney toxicity is 9.8 Gy [17]. Patients of primary gastric lymphoma (PGL) have good prognosis; thus, they would be long-time survivors. Therefore, from the standpoint of keeping adequate renal functions in the long term, the dose to the kidneys should be kept even lower than 15–18 Gy. Further study of the DVHs of the kidney and liver associated with long-term adequate kidney and liver functions is needed. It would also be beneficial to investigate whether the half-beam method or IMRT are more suitable for treatment of PGL patients.

## CONCLUSION

This study shows an advantage for the half-beam method and IMRT over 3DCRT in the treatment of post-chemotherapy gastric DLBCL in cases where there is a large overlap between the kidney and the PTV.

## CONFLICT OF INTEREST

Part of this study was financially supported by the Cancer Research Development Fund of the National Cancer Center (26-A-18).

## FUNDING

Funding to pay the Open Access publication charges for this article was provided by Cancer Research Development Fund of National Cancer Center (26-A-18).
